# Through the looking glass: ethical considerations regarding LLM-induced hallucinations to medical questions

**DOI:** 10.3389/fdgth.2026.1736616

**Published:** 2026-03-13

**Authors:** Zisis Kozlakidis, Tracy Wootton, Michaela Th. Mayrhofer

**Affiliations:** 1 International Agency for Research on Cancer, World Health Organization, Lyon, France; 2 Institute of Human Genetics, Medical University of Innsbruck, Innsbruck, Austria; 3 Papillon Pathways e.U., Landskron, Austria

**Keywords:** artificial intelligence, dynamic framework, ethical accountability, ethics, hallucinations

## 1 Introduction

Large language models (LLMs) are artificial intelligence (AI)-based systems which have been trained on extensive records derived from articles, books and other internet-based content (typically from a mix of both publicly available and proprietary content) (1). LLMs have become rapidly embedded in digital health ecosystems, promising to augment healthcare delivery, research, and patient engagement (2). Their ability to generate coherent, human-like responses positions them as transformative tools for health information dissemination, clinical decision support, and patient self-management. Yet, their probabilistic foundations carry inherent limitations: they are prone to “hallucinations,” the confident presentation of factually incorrect or biologically implausible outputs (3). While such hallucinations may be benign in creative domains, akin to the favourite book by Lewis Carroll ‘Through the looking glass’, in healthcare the consequences of such hallucinations can be profound. This opinion manuscript examines the key ethical and practical implications of such hallucinations, with particular attention to accountability, transparency, and the shared responsibilities of developers, deployers, and end-users.

As a general approach, LLMs generate responses by predicting likely word sequences rather than assessing factual accuracy. Therefore, there are several technical reasons that may affect hallucinations. The primary one is the biases in training data, where for example a model would assume the universality of a pattern, simply because it occurred frequently within a given training dataset. Moreover, when datasets are incomplete or contain lots of noise such as inaccuracies or contradictions, LLMs can learn correlations across these sources, but when asked to reconcile them, they may synthesize a ‘fictional’ middle ground, opt for a wrong pattern observed in the noise, and thus produce authoritative statements even when no reliable data exists. If a model is trained without explicit grounding in fact-checking or truthfulness, hallucination risks increase, because the model is never penalised for inventing, and only rewarded for coherence and diversity (4).

In turn in clinical practice, this can result in plausible but incorrect clinical content, ranging from misdescriptions of symptoms to fabricated treatment guidelines—reflecting the collective accuracy of symptoms descriptions in the harvested databases (5). These risks are amplified in medicine where additional to potential misdescriptions, misinformation can undermine evidence-based practice, confuse patients, or—in the worst cases—cause harm. However, not all hallucinations carry the same weight. Unlike overtly dangerous outputs, which may be swiftly identified and corrected be experienced healthcare professionals, subtler hallucinations can often persist undetected. These smaller inaccuracies accumulate in public discourse, shaping patient perceptions and subtly eroding trust in digital health over the longer-term. Their collective impact is nontrivial: while each instance may appear harmless, the long-tail effect threatens the integrity of health information ecosystems.

## 2 Ethical dimensions: who bears responsibility?

The central ethical questions revolve on fairness and accountability: who is responsible for harm caused by hallucinations? From a technical perspective, accountability may be understood solely as the need for ensuring quality on the algorithm performance and the protection over the data. However, the AI impacting an individual patient is integrated within the healthcare system. Thus, accountability should be viewed through a more comprehensive framework, able to accommodate such a complexity at the level of the product, the process and the actual decision (6). Five major stakeholder groups warrant consideration:

1. **Developers**—Engineers and companies that design LLMs carry ethical duties to minimize hallucination risk through technical safeguards, rigorous testing, and transparent disclosure of limitations. Steps have been taken to this direction with testing options emerging during LLM development (7).
2. **Deployers**—Institutions and platforms integrating LLMs into health systems have the ethical imperative to ensure (as with any equipment or software) that contextual validation, risk assessment, and alignment with clinical standards take place to pre-defined local and national standards (8). Furthermore, the implementation of such systems touches the principle of fairness, so that any improvements in patient service are as equitable as possible.
3. **End-users**—Both clinicians and patients using these systems are anticipated to exercise critical judgment, recognizing that AI outputs are advisory, not authoritative (9). However, to do so, clinicians and patients alike need to acquire sufficient digital health literacy and be educated to use such systems to best effect (10).
4. **Publishing platforms—**Publishing platforms for international scientific journals so far face a double challenge of promoting accurate peer-reviewing for LLMs presented as part of submitted manuscripts (11), as well as potentially adopting LLM as an aid for the peer review process itself (12). The ethical perspective here, is this of trust, and how to maintain the trustworthiness of the wave of new publications that results from the rapid technological advancement.
5. **Regulators—**The efforts to regulate effectively and thereafter maintain control over regulated domains, e.g., in the European Union (EU) with the AI Act (13), the China Cybersecurity Law (14) and the UK pro-innovation, context-specific approach, underscore the regulatory urgency of implementing national and international regulations (15). However, even more effective would be the creation of an aligned regulatory framework in the near future (16).

The ethical complexity lies in the interaction of these key stakeholder groups and their distinct roles. While end-users cannot be expected to independently audit every claim (and due to the technological complexity objectively cannot do so), neither can developers disclaim all liability. Moreover, there is an acute need to translate abstract AI ethical principles into actionable frameworks within healthcare, where the social good is a critical underpinning. This urgency is proportionate to the risk, as AI-driven technologies have the potential to be both powerful and disruptive once implemented (17). Publishing platforms need to create consensus guidelines for the review and assessment of LLMs both as in-house tools, as well as engines driving scientific discovery. In a similar manner, solid assessment criteria need to be developed and implemented by the deployers. Thus, it is the strong opinion of the authors that a dynamic, multi-stakeholder ethical accountability framework is essential.

## 3 Dynamic ethical accountability

Transparency is at the foundation of ethical accountability, both a technical and ethical imperative in the healthcare-related LLM implementation, as -at a minimum- users must be clearly informed about the probabilistic nature of LLM outputs and their limitations in terms of generating hallucinations, and in turn be able to inform patients’ and clinicians’ decisions (18). Several AI guidance documents have been published recently on this basis and refined by many actors (19–21). Theoretically, this can include the disclosure of data provenance (esp. for training data), the communication of uncertainty (e.g., with confidence scores), and the clear indication throughout that any outputs are non-substitutive for medical advice. In this way, the discourse shifts from blind reliance to informed engagement (22) and allows clinicians and patients to situate LLM-generated information within evidence-based frameworks. However, this is an ideal and static scenario, that anticipates high levels of digital literacy and understanding from all parties, as well as the ability to identify technological nuances. However, in the case of the LLM rapid technological development and logarithmic increase of related publications, it is not sufficiently practical. Thus, the authors support the need for a dynamic ethical accountability framework.

In this model,

1. The developers ensure ethical governance, transparency and safety by-design; potential quantifiable metrics can include the number/proportion of data sources with fully documented provenance, and/or for existing LLMs the % of new model versions passing pre-deployment safety tests (for both ideally at an eventual 100%)
2. The deployers operationalise safety and fairness in real-world use; potential metrics can include a report for the number of LLM safety or fairness incidents reported per 1,000 clinical interactions (with a targeted decrease by 30–50% within 3 years) and/or a report for the number of clinical departments using the LLM that fully implement required safety policies;
3. The end-users are co-creators of consent models and define acceptable, even personalised, risk thresholds; potential metrics can include a published number of clinical pathways that include LLM-specific informed consent steps, complemented by the reported increase in clinician competency scores in AI/LLM literacy testing (with a targeted improvement over time, relevant to the institutions);
4. Publishing platforms provide oversight of the moving field and emerging requirements; potential metrics can include the average time taken to issue updated editorial requirements following major AI regulatory changes (eventual target: <6 months); and
5. Regulators (including ethics boards) provide arbitration and are able to service legal enforceability. Potential metrics for the latter can include publishing the median time from ethical concern submission to regulatory decision, complemented by the % of healthcare deployers certified as compliant with updated LLM regulations (with the eventual aim being over 85% at any point in time).

The dynamic features of such a model need to include key components that allow it to re-define parameters as the technology progresses. For example, these can be regular “ethical stress tests”, e.g., simulation tests whether systems can withstand ethical risks such as biased LLM outputs and/or data misuse. The implementation of “safety override mechanisms”, where any stakeholder can flag ethical concerns and request support or -at worst case scenario- stop temporarily any deployment. Moreover, scaled responsibility agreements can be incorporated, with distinct expectations for large AI developers (e.g., transparency audits, bias mitigation, public reporting), mid-level hospitals (e.g., local oversight boards, community engagement), and smaller clinics/personal users (e.g., reduced, streamlined obligations but mandatory escalation channels). Additionally, the incorporation of neuro-rights into the framework is essential to safeguarding patients’ cognitive integrity and mental privacy in clinical contexts. As hallucinated outputs may inadvertently distort a patient's perception of reality, LLM deployment in healthcare should explicitly account for the potential impact on these emerging rights (23). Accordingly, any ethical responsibility framework for medical AI must include mechanisms and protocols that prevent cognitive manipulation and ensure robust protection of users’ mental privacy. For example, one such mechanism can be a “mental privacy protection protocol”, where conversational data involving thoughts, emotions, or sensitive cognitive states is processed under a strict no-inference rule, prohibiting the model from generating speculative interpretations about a patient's intentions, psychological profile, or future behaviour (24). In practical terms, access to such data can be tightly controlled and anonymised, ensuring that the system does not intrude upon or manipulate a patient's mental perceptions.

While the above proposal may seem challenging to implement to healthcare, it is worthwhile stressing that they exist in other equally complex and technologically driven fields of activity. Specifically, within the financial sector regular scenario simulations test whether systems can withstand different risks, and also partly adopted within healthcare as table-top exercises on infectious disease outbreaks (25). Scaled responsibility agreements have been implemented on climate-related activities and governance models, while the safety override mechanisms is a core tenet of the aviation industry (26), where any stakeholder can “pull the brake” on deployment if safety concerns are raised.

For example, the practical deployment of an LLM tool in oncology based on the dynamic ethical accountability framework, will require that before deployment the industry conducts transparency audits and hallucination detection tests and regulators approve conditional use; during deployment clinicians monitor outcomes and patients have clear digital and open access channels for feedback or opt-out; and after deployment regular ethical stress tests take place, using the real-world data, with responsibilities rebalanced depending on findings (e.g., if algorithmic bias is discovered, greater burden falls on developers). More importantly, if any risks emerge, any stakeholder (clinician, ethics board, patient/patient representative) can trigger a review (“safety override”). While individual elements of this approach may be in existence already and can draw upon recent recommendations and guidelines developed by the above-mentioned stakeholders, they are—to date—not provided within a coherent, unified framework and focus on a risk-based, rather than a dynamic accountability approach.

However, it is important to note that countries with less developed legal and regulatory frameworks—often, but not always in resource-restricted settings- have the potential to face acute challenges managing LLM hallucinations in clinical settings, as this challenge sits at the intersection of technical opacity, limited regulatory capacity, and fragile health systems. Precisely because many regulatory bodies can lack technical expertise and resources to assess model provenance, evaluate validation studies, or mandate independent transparency audits; this gap makes it difficult to determine liability when an LLM gives a confidently stated but incorrect diagnosis or treatment suggestion (27). Additionally, there is the potential for language and cultural mismatch between globally trained models and local practice increases the risk of errors: regionally tailored initiatives such as Latam-GPT underscore the need for local data and evaluation precisely because English-centric models can underperform in non-Anglophone clinical contexts (28). Equally as important is that many LMIC health systems frequently lack robust clinical-incident reporting and resources for independent replication studies — mechanisms essential to detect, quantify, and remediate hallucination-driven harms. Some promising frameworks and scoring systems have been developed recently to measure hallucination frequency and citation authenticity (e.g., the Reference Hallucination Score) (29), however, they still require regulatory uptake and capacity to operationalize, raising the perennial question on the relative speeds between technological development and regulatory guidance (30).

## 4 Conclusion

It is our opinion that ethical stewardship of LLMs in digital health requires collective responsibility. Equally, public health systems must anticipate the societal implications of cumulative hallucinations—or even worse, potential deliberate data poisoning attempts (31)—ensuring that vulnerable individuals/populations are not disproportionately affected by misinformation. Importantly, guidelines should evolve dynamically alongside the technology, incorporating real-world monitoring and feedback to capture unanticipated risks. This evolution requires flexibility, so that guidelines adapt and implement as technology and risks evolve; inclusivity, so that all stakeholders have voice and power; fairness, where larger actors carry proportionally greater ethical duties; and finally, resilience, so that built-in learning loops prevent repeating failures.

Current ethical frameworks in digital health—such as the principles of beneficence, non-maleficence, and justice—remain relevant but require reinterpretation in the age of generative AI. Lessons can be drawn from prior digital transformations, including electronic health records and clinical decision support systems, where overreliance on algorithmic recommendations sometimes led to errors. Regulatory and professional bodies must establish new guidelines tailored to LLMs, emphasizing accuracy, explainability including explaining what hallucinations are and their potential impact, and recourse for error correction. As digital health moves further “through the looking glass” of LLM integration, the community must ensure that innovation does not outpace ethical reflection. Rather than asking *who alone is accountable*, the more constructive question is: *how can accountability be distributed fairly and effectively across the ecosystem*? The dynamic ethical accountability framework offers such an approach.
